# Techno-functional, nutritional, and health-promoting properties enhancement of mopane worm and orange-fleshed sweet potato flour blends via ultrasonication and controlled fermentation

**DOI:** 10.3389/fmicb.2025.1688648

**Published:** 2025-12-02

**Authors:** Mpho Brian Molimi, Oluwafemi Ayodeji Adebo

**Affiliations:** Centre for Innovative Food Research (CIFR), Department of Biotechnology and Food Technology, Faculty of Science, University of Johannesburg, Doornfontein, Johannesburg, South Africa

**Keywords:** edible insects, functional food ingredients, health, nutrition, pasting properties, zero hunger, good health and well being

## Abstract

This study investigated the impact of fermentation and ultrasonication on biochemical, nutritional, and health-promoting properties of mopane worm (MP) and orange-fleshed sweet potato (OFSP) flours, followed by nutritional, health-promoting, pasting, and thermal properties of their subsequent blends, derived from three MP: OFSP blending ratios (60:40, 55:45, and 45:55) for each processing technique. Respective flour was fermented using starter culture (mesophilic lactic acid bacteria) for 48 h at 35 °C and ultrasonicated at 500 W, 20 kHz for 5 min. Both processes significantly reduced pH and increased the total titratable acids (TTA) of all flours. Increments in protein, ash, and total flavonoid content (TFC) were observed in all fermented flours, while ultrasonicated flours exhibited elevated fiber and total phenolic content (TPC). Unlike ultrasonication, fermentation reduced the 2,2-Azinobis (3-ethyl-Benzothiazoline-6-sulfonic acid) (ABTS) of all flours. After blending processed MP and OFSP flours, there were beneficial modifications in the *in vitro* starch digestibility of their resultant blends. For instance, the addition of 60% MP significantly reduced (*p* < 0.05) rapidly digestible starch (RDS) in fermented blends, while ultrasonicated blends had lower total digestible starch (TDS). The TFC, TPC, and ABTS of all the blends varied from 3.83 to 5.06 mgQE/g, 1.90–2.76 mgGAE/g, and 52.93–61.03%, respectively. Higher peak viscosity in fermented blends reflects good water-binding capacity and the ability to produce a highly viscous gel. Intrinsic alterations in thermal properties were observed in fermented blends, involving the reduction of onset and peak temperatures. The present study revealed that both fermented and ultrasonicated blends containing 60% MP and 40% OFSP flour had beneficial complementary properties, which may be excellent for the development of novel food products with improved health and nutritional advantages.

## 1 Introduction

Chronic illnesses (diabetes, heart diseases, cancers, etc.), caused by poor dietary habits, are major public health concerns worldwide, posing a significant health threat, especially in aged individuals (Baker et al., 2022). The high cost of pharmaceuticals, as well as their side effects, has led researchers to explore new possibilities, such as functional foods for prevention and management of diet-related issues, including chronic diseases (Pearce et al., 2017; Adefegha, 2018; Oladimeji and Adebo, 2024). Additionally, are the challenges of protein-energy malnutrition and general burden of nutritional deficiencies, which are higher in low-income countries (Bayati et al., 2025). Studies have also shown that individuals in such countries are less likely to consume foods containing the right combination of nutrients (Darmon and Drewnowski, 2008; Fisher et al., 2022). With the increased cases of obesity, wasting, stunting, underweight, and nutritional deficiencies, the development and availability of nutrient-dense and functional foods have become increasingly relevant (Chinma et al., 2022a; Jimoh and Adebo, 2025; Moazzam et al., 2025). Functional foods offer additional health benefits that can positively influence the immune system and physiology of the human body (Topolska et al., 2021). Basically, food is deemed functional if it contains, fortified or supplemented with health-improving ingredients, such as bioactive compounds, vitamins, minerals, including probiotics and prebiotics, and also when all antinutrients and other harmful compounds are eradicated (Alongi and Anese, 2021; Martirosyan et al., 2022). Most underutilized food sources, such as fruits, vegetables, legumes, roots, tubers, and insects, are packed with enormous amounts of nutrients and biologically active constituents with functional roles (Okigbo and Anyaegbu, 2021; Ballini et al., 2023; Knorr and Augustin, 2025; Saeed et al., 2025).

OFSP (*Ipomoea batatas*) is a biofortified underutilized tuberous crop, valued for high amounts of beta-carotene and a broad range of bioactive compounds, such as flavonoids, phenolic acids, including vitamin C, vitamin D, and vitamin B complexes (Oloniyo et al., 2021; Kewuyemi and Adebo, 2024; Knorr and Augustin, 2025). Studies have shown that blending orange-fleshed sweet potatoes with other food sources, such as wheat (Korese et al., 2021), amaranth (Giri and Sakhale, 2021), maize (Baah et al., 2022), cowpea (Kewuyemi et al., 2025) etc., enhance the phenolic compounds and antioxidant activities of the final blends. However, the presence of antinutritional factors like tannins, cyanides, phytic acids, and oxalates in sweet potatoes severely affects the bioavailability and bioaccessibility of the crop’s nutritional value (Amagloh et al., 2021; Bhadram et al., 2024). MP (*Gonimbrasia belina*), on the other hand, are wild edible insects, mostly found in Southern African countries, where mopane trees are prevalent (Nantanga and Amakali, 2020; Ruzengwe et al., 2023). These insects comprise a high protein content ranging between 60 and 70% (Maleke et al., 2024). The utilization of MP in food-to-food fortification has gained momentum over the years. Today, it is evident that incorporating MP in food enhances its protein content (Gabaza et al., 2018; Moleha and Mphosi, 2019; Vanqa, 2022; Mashau et al., 2024). Despite high protein content, mopane worms often exhibit poor digestibility and performance in real food applications.

Therefore, processing techniques such as fermentation and ultrasonication that can beneficially modify the nutritional value, techno-functional attributes, and bioactive compounds of underutilized food sources are vital for driving innovation in the development of novel healthy food products (Knorr and Augustin, 2025; Mahlanza et al., 2025; Saeed et al., 2025). Fermentation is a traditional technique that uses microbial enzymatic actions to induce changes in the characteristics of food materials (Mudau and Adebo, 2025). Solid-state fermentation (where microorganisms grow in solid organic media having less or almost no free-flowing water) and submerged fermentation (where the growth of microorganisms occurs in liquid media) are two fermentation methods used for food processing (Doriya et al., 2016; Yafetto, 2022). On the contrary, ultrasonication is a novel food processing technique that has gained recognition due to its low cost, eco-friendliness, and its ability to improve food safety and quality (Mudau and Adebo, 2025). During ultrasound processing, high-frequency sound waves are transferred to the substrate through acoustic cavitation, which forms a rapid collapse and expansion within the substrate, leading to changes in the physicochemical, structural, and techno-functional characteristics (Cui and Zhu, 2020).

The application of these processes in MP and OFSP is necessary for improving their health benefits, functionality, digestibility, as well as their safety. Kewuyemi and Adebo (2024) reported elevated levels of protein, hemicellulose, resistant starch, and a broad range of phenolic compounds such as trans-ferulic acid, p-coumaric acid, sinapic acid, vanillic acid, luteolin, and taxifolin in OFSP after solid-state fermentation, including a reduced content of tannin and oxalate. Ultrasound processing in sweet potato induced alterations in starch granules and particle size, leading to improved starch digestibility, as well as pasting and thermal characteristics (Cui, 2021). In MP, the influence of these processing techniques was investigated by Maleke et al. (2024). Both processes improved the protein, fiber, and TFC of MP flour, but their levels were higher in the fermented sample. Moreover, ultrasonication increased the water and oil holding capacity of the MP, summing up the distinct benefits of each processing technique. Despite the improved nutrition, health, and functionality of fermented and ultrasonicated MP and OFSP, little is known regarding their blends. Furthermore, their blend performance in food processing applications is unknown. Therefore, the current study aimed to investigate the impact of fermentation and ultrasonication on the nutritional, health-promoting, pasting, and thermal properties of MP and OFSP flour blends.

## 2 Materials and methods

### 2.1 Raw materials

Raw kara OFSP was procured at Woolworths (Johannesburg, South Africa), and dried MP (*Gonimbrasia belina*) at street vendors (Krugersdorp, South Africa). The OFSP (*Ipomoea batatas*) was stored in a refrigerator and MP at room temperature until needed for flour processing. The freeze-dried starter (mesophilic lactic acid bacteria) culture used was acquired from CHR HANSEM, Denmark. Sunflower oil (d’lite) used was sourced from Shoprite (Johannesburg, South Africa).

### 2.2 Flour processing

**Figure 1:**
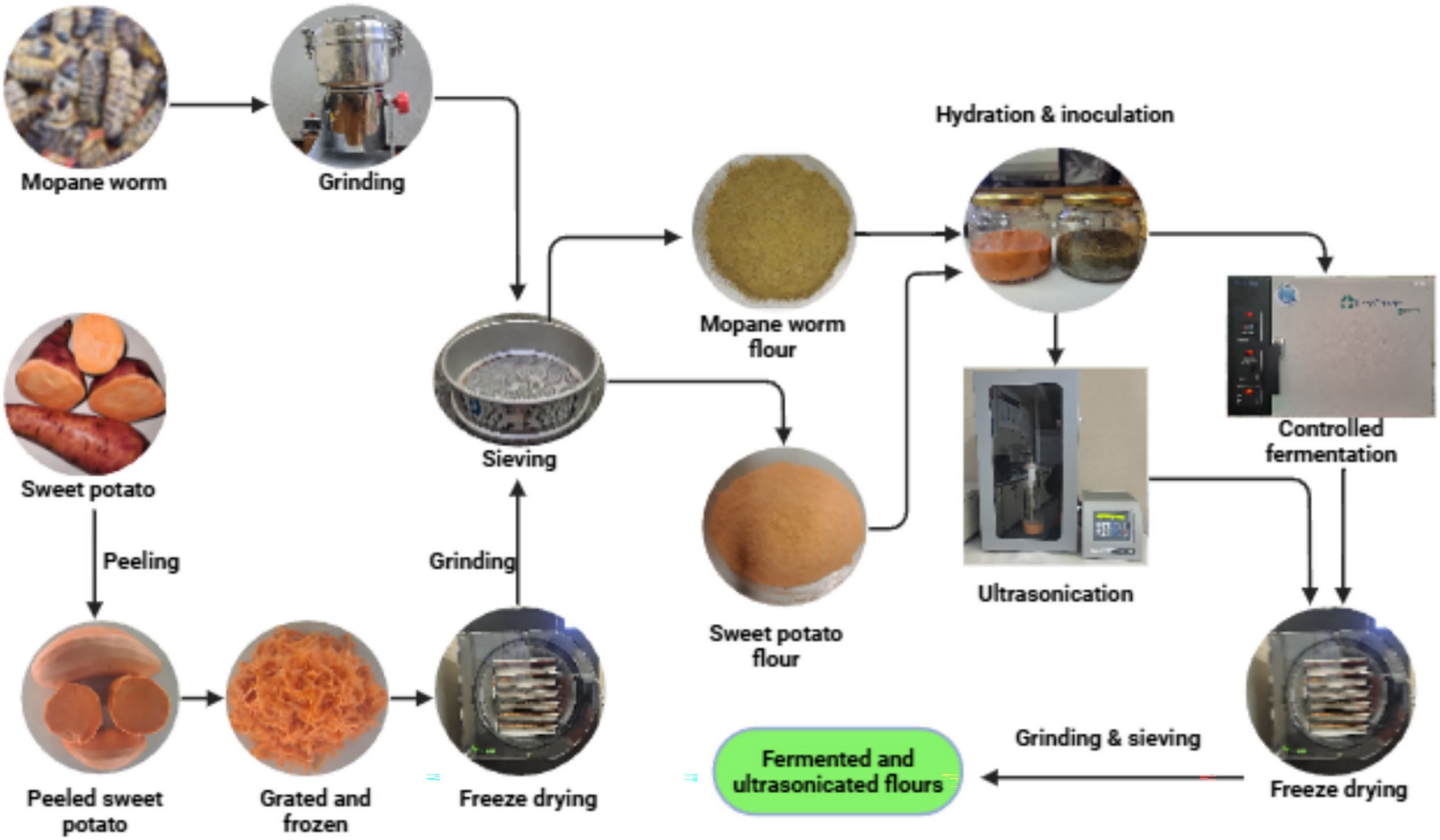
Process flow of MP and OFSP flours.

The processing flow chart of OFSP and MP flours is illustrated in Figure 1. OFSP was peeled, grated, frozen, and freeze-dried (Harvest Right, Salt Lake City, USA). The freeze-dried sample was ground to obtain OFSP flour. The MP flour was acquired by grinding dried procured worms. All the flours were sieved using a 250 μm sieve separately and stored in plastic ziplock bags until needed for further investigations.

### 2.3 Controlled fermentation

The raw OFSP and MP flours were fermented using a previously defined procedure (Kewuyemi and Adebo, 2024). A 100 g of flour, 100 mL of sterile water, and 0.40 g of starter culture were uniformly mixed and allowed to ferment in an incubator (LABOTEC, Midrand, South Africa) for 48 h at 35 °C. The resultant sourdoughs were freeze-dried, milled, and sieved (250 μm) to obtain fermented flours.

### 2.4 Ultrasonication

Ultrasonication was performed using a previously modified procedure (Maleke et al., 2024). Briefly, 100 mL of sterile water and 30 g of flour were mixed and treated with a probe ultrasonicator (VCX 500, New York, USA) using 500 W 20 kHz frequency, 15 s on 5 s off pulse rate, 50% amplitude for 5 min. The recovered samples were frozen, freeze-dried, ground, and passed through a 250 μm sieve.

### 2.5 Biochemical properties

The total soluble solids (TSS) of flours were evaluated using and refractometer (HANNA, Nusfalau, Romania), and pH using a pH meter. The supernatant obtained by centrifuging the mixture of 2.5 g of flour and 25 mL of purified water at 4,000 rpm for 10 min at 0 °C, was used for total titratable acids (TTA) determination. About 10 mL of the supernatant was titrated with 0.1 N NaOH to pH 8.3, and the results were expressed as mL/g as per the procedure adopted by Kewuyemi and Adebo (2024).

### 2.6 Nutritional composition

The proximate composition based on crude fiber, ash, protein, and moisture content was evaluated according to (AOAC, 2006) method 990.03, 934.01, 978.10, and 925.09, respectively. The results were expressed as percentages (%).

### 2.7 Techno-functional properties

#### 2.7.1 Oil and water absorption capacity

The oil absorption (OAC) and water absorption (WAC) capacity of the flours were determined following a modified procedure by Mudau et al. (2022). 1 g of flour and 10 mL of sunflower oil/purified water were added to centrifuge tubes. The suspension was vortexed (K-550-GE, Florida, USA), and centrifuged (Eppendorf 5702R, Midrand, South Africa) at 3,000 rpm for 10 min. Thereafter, the supernatant (oil/water) was decanted, and the tube was inverted for 1 h to drain out the excess oil/water. The OAC of flours was calculated using Equation 1. After removing the excess water, the paste was dried at 60 °C for 5 h, and the WAC of samples was calculated using Equation 2.


$$\mathrm{OAC}(g/g)=\frac{\text{Weight of oil containing sample}-\text{Weight of sample}}{\text{Weight of sample}}$$ (1)

$$\mathrm{WAC}(g/g)=\frac{\text{Weight of sample}-\text{Weight of dried sample}}{\text{Weight of sample}}$$ (2)

#### 2.7.2 Solubility and swelling power

The solubility and swelling power (SP) of the flours were evaluated following a modified procedure by Zhang et al. (2019). Approximately 1 g (W_0_) of flour and 10 mL of purified water were vortexed (K-550-GE, Florida, USA). The suspension was then incubated in a water bath (Labcon, Johannesburg, South Africa) for 30 min at 85 °C, cooled at room temperature, and centrifuged (Eppendorf 5702R, Midrand, South Africa) at 3000 rpm for 10 min at 0 °C. The supernatant was decanted into a 50 mL glass beaker of known weight and dried to a constant weight (W_1_). The remaining precipitate was weighed (W_2_), and the solubility and SP of the samples were calculated using Equations 3, 4, respectively.

$$\text{Solubility}\;(\%)=\frac{W_{1}}{W_{0}}\times 100$$ (3)

$$\mathrm{SP}\;(g/\mathrm{ml})=\frac{W_{2}}{\left(W_{0}(1-\frac{S}{100}\right)}$$ (4)

### 2.8 Health-promoting properties

#### 2.8.1 Acidified methanolic extraction

Approximately 0.50 g of the sample was added to a centrifuge tube, followed by 5 mL of 80% methanol containing 1% HCl. The mixture was then vortexed (K-550-GE, Florida, USA) and water bath sonicator (ARGO LAB, Via della Meccanica, Italy) for 1 h at 4 °C. After sonication, the contents were centrifuged for 10 min at 0 °C and 4,300 rpm. The supernatant was filtered and decanted into a clean centrifuge tube as a sample extract.

#### 2.8.2 2,2-Azinobis (3-ethyl-Benzothiazoline-6-sulfonic acid) assay

The 2,2′-Azinobis (3-ethyl-Benzothiazoline-6-sulfonic acid) (ABTS) assay was conducted following the procedure adopted in a previous study by Kewuyemi et al. (2023). The prepared ABTS (7.6 mM) and K_2_S_2_O_8_ (2.6 mM) solutions were combined and incubated in a dark space for 16 h. Next, 1 mL of the mixture was diluted in 60 mL of distilled water to form a working solution. Thereafter, 20 μL of sample extract was added to a microplate, followed by 200 μL of working solution. Samples were assayed using an Accuris microplate reader (MR9600, Jersey City, USA) at 734 nm, and the results were expressed as inhibition percentage (%).

#### 2.8.3 Total flavonoid content

The solutions of 1.25% AlCl_3_, 2.5% NaNO_3_, 2% NaOH, and varying concentrations of quercetin were prepared. In the microplate, 10 μL of sample extract and standard solution were added separately, followed by 30 μL of NaNO_3_ and AlCl_3_, and 100 μL of NaOH. Samples were incubated for 30 min at room temperature in a dark space. An Accuris microplate reader (MR9600, Jersey City, USA) was used to read the samples at 450 nm, and the results were expressed as milligrams of quercetin equivalent per gram (mgQE/g) as per the previously described procedure (Maleke et al., 2024).

#### 2.8.4 Total phenolic content

Solution of 7.5% Na_2_CO_3_, 6.7% Folin–Ciocalteu, and varying concentrations of gallic acid were prepared. Then 10 μL of sample extract and standard solution were added to a microplate separately, followed by 50 μL of Folin–Ciocalteu solution, and Na_2_CO_3_. The microplate was incubated in a dark space for 30 min at room temperature, and an Accuris microplate reader (MR9600, Jersey City, USA) was used to assay the samples at 750 nm. The results were expressed as milligrams of gallic acid equivalent per gram (mgGAE/g) as per the previously described procedure by Maleke et al. (2024).

### 2.9 Flour blending ratios

Processed flours were blended into three MP: OFSP ratios (60:40, 55:45, 55:45), for each processing technique as shown in Table 1. Blends were mixed thoroughly and subjected to five cycles of sieving using a 250 μm sieve to eliminate foreign objects and yield better homogeneity.

**Table 1:** Composite ratios of raw and processed MP and OFSP flours.

Flour		UMP – 60	UMP – 55	UMP – 45	FMP – 60	FMP – 55	FMP – 45
Ultrasonicated	Mopane worm (%)	60	55	45	0	0	0
	Sweet potato (%)	40	45	55	0	0	0
Fermented	Mopane worm (%)	0	0	0	60	55	45
	Sweet potato (%)	0	0	0	40	45	55

UMP – 60, ultrasonicated 60% MP and 40% OFSP blend; UMP – 55, ultrasonicated 55% MP and 45% OFSP blend; UMP – 45, ultrasonicated 45% MP and 55% OFSP blend; FMP – 60, fermented 60 and 40% OFSP blend; FMP – 55, fermented 55% MP and 45% OFSP blend; FMP – 45, fermented 45% MP and 55% OFSP blend.

### 2.10 *In vitro* starch digestibility

The rapidly digestible starch (RDS), slowly digestible starch (SDS), resistant starch (RS), and total digestible starch (TDS) in the composite blends were evaluated following the procedure adopted in a previous study by Kewuyemi and Adebo (2024) using a Magazyme kit. Approximately 0.5 g of sample was added to a 250 mL Fisherbrand bottle, followed by adding 15 glass balls, 1.0 mL of 95% ethanol, and 35 mL of sodium maleate buffer solution (50 nM, pH 6) sequentially. The bottles were placed in a shaking water bath (LABOTEC, Midrand, South Africa) set at 170 rpm and 37 °C, followed by the addition of 5 mL of amyloglucosidase solution 5 min later. Then 1 mL of aliquot was removed from the bottles at 20, 120, and 240 min, and transferred into centrifuge tubes, followed by the addition of 20 mL of acetic acid solution (50 nM). Thereafter, 2 mL of the aliquot was transferred into a microfuge tube and centrifuged (Appendorf, Hamburg, Germany) at 13,000 rpm for 5 min. Then 1 mL of centrifuged aliquot and amyloglucosidase solution were added into a 15 mL centrifuge tube, mixed well, and incubated at 50 °C for 30 min. Finally, the amount of glucose released after 20 min (RDS), 120 min (SDS), and 240 min (RS and TDS) of digestion was evaluated using the GOPOD reagent and assayed using an Accuris microplate reader (MR9600, Jersey City, USA) at 510 nm.

### 2.11 Pasting properties

Based on the modified procedure by Kewuyemi et al. (2024) using a rheometer (Anton Paar MCR 72, Graz, Austria), the pasting properties of MP and OFSP flour blends were evaluated. Precisely, a mixture of a sample (5 g) and water (±25 mL – based on moisture content) was placed inside the system using a measuring cup. Samples were analyzed at 50 °C with a revolving probe set at 160 rpm. The results were expressed as peak viscosity (mPa s), peak time (min), breakdown viscosity (mPa s), setback viscosity from peak (mPa s), setback viscosity from trough (mPa s), final viscosity (mPa s), and pasting temperature (°C).

### 2.12 Thermal properties

The thermal properties of samples were determined following a modified procedure by Mudau and Adebo (2025), using a differential scanning calorimeter (DSC) (Mettler-Teledo, Greifensee, Switzerland). Approximately 10 mg of the sample was added into an aluminum pan followed by 30 μL of distilled water. The pan was sealed and left to hydrate for 24 h. The hydrated and reference (empty aluminum pan) samples were heated at the rate of 10 °C/min using a heating range of 25–160 °C. The results were categorized as onset temperature (T_O_), peak temperature (T_P_), conclusion temperature (T_C_), and enthalpy change (∆H).

### 2.13 Statistical analysis

Analysis of variance (ANOVA) was adopted using SPSS software (IBM SPSS Statistics Version 22, New York, USA), and the significant differences were decided at a 95% (*p* < 0.05) confidence interval. Experiments were conducted in triplicate, and the means were separated using the Duncan multiple range test.

## 3 Results and discussion

### 3.1 Biochemical properties of processed flours

**Figure 2:**
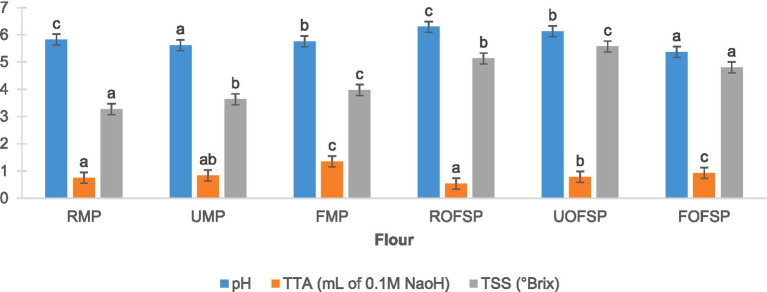
Biochemical properties of processed MP and OFSP flour. Results are triplicate mean ± standard deviation (SD) with different superscript letters on each bar signifying significant differences (*p* < 0.05): TTA, total titratable acid; TSS, total soluble solids; RMP, raw mopane worm flour; UMP, ultrasonicated mopane worm flour; FMP, fermented mopane worm flour; ROFSP, raw orange-fleshed sweet potato flour; UOFSP, ultrasonicated orange-fleshed sweet potato flour; FOFSP, fermented orange-fleshed sweet potato flour.

The biochemical properties (pH, TTA, and TSS) of fermented and ultrasonicated MP and OFSP flours are illustrated in Figure 2. Interestingly, the pH of raw MP and OFSP flours was comparable to that of Maleke et al. (2024) and Kewuyemi and Adebo (2024), respectively. Fermentation significantly (*p* < 0.05) reduced the pH of both flours as anticipated, due to the break down of sugars and other food constituents into organic acids. On the other side, Ultrasonication reduced the pH of both flours, but a significant reduction (*p* < 0.05) was observed in MP flour. These observations are in agreement with those reported by Roobab et al. (2024) and may be interlinked with acidifying effects (disruption of cellular structures and release of organic acids) of ultrasonic cavitation. However, in a previous study (Maleke et al., 2024), an increase in the pH of MP flour after ultrasonication was observed. The type of ultrasonic equipment used and the sample particle size might have brought these varying observations between the current study and that of Maleke et al. (2024). A declining pH in both fermented and ultrasonicated MP and OFSP flours correlated with an increasing TTA. According to Adebo et al. (2021), this phenomenon in fermented samples demonstrates the effect of microbial activity, which could be beneficial against pathogens in fermented foods. Both fermentation and ultrasonication significantly (*p* < 0.05) increased the TSS of MP flour, and this trend was previously reported in finger millet flour (Mudau et al., 2024). These results contribute to the existing knowledge that both processes have different influences on different food materials.

### 3.2 Selected nutritional composition of raw and processed flours

Table 2 shows the nutritional composition of raw and processed MP and OFSP flours. The moisture content of raw MP and OFSP flour was 6.58 and 10.67%, respectively. Fermentation significantly (*p* < 0.05) increased the ash content of both flours, aligning with the findings of Kewuyemi and Adebo (2024). Ultrasonication increased the fiber content of all investigated flours, and it is known that the dietary fiber of food materials tends to increase after ultrasonic processing (Spotti and Campanella, 2017). In contrast, fermentation reduced the fiber content of OFSP, yet an opposite trend was reported by Kewuyemi and Adebo (2024). These observations may be ascribed to the different OFSP varieties or harvesting time. It is noteworthy that certain microbial strains, particularly lactic acid bacteria, are capable of breaking down dietary fiber and cell wall components of plant-derived food sources, which could lower their fiber content (Fujimori, 2021; Kitessa, 2024). Significant (*p* < 0.05) reduction in the protein content of ultrasonicated MP flour contradicts the findings of Maleke et al. (2024), possibly due to shorter ultrasonic treatment (5 min) adopted in the present study. Ultrasonic treatment has been reported to influence the protein structure and functionality in food materials by initiating or breaking molecular crosslink and hydrogen bonding interactions, depending on the nature of the food matrix. In some instances, amino acids undergo partial hydrolysis when exposed to ultrasonic cavitation, leading to reduced protein content (Wang et al., 2023; Zhang et al., 2023). As anticipated, fermentation increased the protein content of both flours despite non-significant (*p* > 0.05) observations. According to Kewuyemi and Adebo (2024). Elevated protein content in fermented flour is caused by microbial actions that break down complex protein molecules into free and biaccessible amino acids.

**Table 2:** Selected nutritional composition of raw and processed MP and OFSP flours.

Sample	Moisture (%)	Ash (%)	Fiber (%)	Protein (%)
Mopane worm				
RMP	6.58^b^ ± 0.05	10.76^c^ ± 0.05	6.70^a^ ± 0.29	56.35^b^ ± 0.85
UMP	9.13^c^ ± 0.08	10.01^a^ ± 0.17	9.35^c^ ± 0.30	54.57^a^ ± 0.83
FMP	5.60^a^ ± 0.15	10.91^b^ ± 0.14	7.34^b^ ± 0.38	57.08^b^ ± 1.48
Orange-fleshed sweet potato				
ROFSP	10.67^b^ ± 0.02	2.38^a^ ± 0.15	4.28^b^ ± 0.04	5.15^a^ ± 0.19
UOFSP	9.46^a^ ± 0.08	2.23^a^ ± 0.09	4.38^b^ ± 0.14	5.05^a^ ± 0.26
FOFSP	11.26^c^ ± 0.18	2.91^b^ ± 0.08	3.56^a^ ± 0.06	6.09^a^ ± 0.23

Results are expressed as triplicate mean ± SD with different superscript letters on each column signifying significant differences (*p* < 0.05). RMP, raw mopane flour; UMP, ultrasonicated mopane worm flour; FMP, fermented mopane worm flour; ROFSP, raw orange-fleshed sweet potato flour; UOFSP, ultrasonicated orange-fleshed sweet potato flour; FOFSP, fermented orange-fleshed sweet potato flour.

Ash content increment leads to increase in mineral content and an indication of the level of mineral composition of the substrates Ash content increment leads to increase in mineral content and an indication of the level of mineral composition of the substrates Ash content increment leads to increase in mineral content and an indication of the level of mineral composition of the substrates Ash content increment leads to increase in mineral content and an indication of the level of mineral composition of the substrates. Ash content increment leads to increase in mineral content and an indication of the level of mineral composition of the substrates.

### 3.3 Health-promoting properties of raw and processed flours

The health-promoting properties of raw and processed MP and OFSP flours are presented in Table 3. Despite non-significant findings (*p* > 0.05) across the TFC of all investigated samples, fermented flours had the highest TFC. In fact, the TFC of FMP and FOFSP were 5.24 mgQE/g and 2.30 mgQE/g, respectively, compared to 4.92 mgQE/g and 2.11 mgQE/g of their respective raw samples. In contrast, ultrasonic treatment induced an opposite effect in the TFC of all flours, which could be associated with the degradation of phenolic compounds either through direct pyrolysis reactions or the formation of radicals, prompted by cavitation bubbles within the sample (Cui and Zhu, 2020; Olatunde et al., 2025). No significant differences (*p* > 0.05) were observed in the TPC of raw and processed flours. However, both processing techniques showed distinct modifications in the OFSP sample. UOFSP had a TPC of 7.95 mgGAE/g, lower compared to 14.98 mgGAE/g in FOFSP, which was the highest. Increments in TFC and TPC of insect and root flour after fermentation have been previously reported (Kewuyemi and Adebo, 2024; Maleke et al., 2024; Mahlanza et al., 2025). Microbial enzymatic activities of mesophilic lactic acid bacteria can facilitate the release of insoluble and bound phenolic compounds during fermentation (Saharan et al., 2017), which might have been the case in the present study. Similarly, ultrasonication increased the TPC of MP flour, aligning with a previous finding (Maleke et al., 2024), and suggested that ultrasonic cavitation effects might have liberated entrapped phenolic acids. Regarding the antioxidant activities based on the ABTS assay, both fermentation and ultrasonication reduced the antioxidant capacity of the OFSP sample, even though the results were statistically insignificant, especially in OFSP flour. A significant reduction (*p* < 0.05) in the ABTS of MP flour after fermentation may be related to the metabolization of some phytochemicals, which could also act as a source of nutrients for microorganisms (da Cruz et al., 2025). High ABTS in UMP signifies optimal human health benefits and the ability to scavenge reactive oxygen species, thereby preventing or reducing the peril of oxidative stress-related complications such as diabetes, inflammation, cancer, and cardiovascular diseases (Lang et al., 2024). The impact of fermentation and ultrasonication on the health-promoting properties of the samples varied greatly depending on the nature of the food material.

**Table 3:** Health-promoting properties of raw and processed MP and OFSP flours.

Sample	TFC (mgQE/g)	TPC (mgGAE/g)	ABTS (% inhibition)
Mopane worm			
RMP	4.92^a^ ± 0.47	9.89^a^ ± 5.87	48.96^b^ ± 3.70
UMP	4.79^a^ ± 0.47	14.93^a^ ± 0.99	51.54^b^ ± 2.93
FMP	5.24^a^ ± 0.53	11.55^a^ ± 6.85	39.90^a^ ± 5.15
Orange-fleshed sweet potato			
ROFSP	2.11^a^ ± 0.02	12.70^a^ ± 2.92	51.81^a^ ± 3.47
UOFSP	2.09^a^ ± 0.05	7.95^a^ ± 5.43	47.58^a^ ± 3.94
FOFSP	2.30^a^ ± 0.03	14.98^a^ ± 2.79	46.95^a^ ± 3.40

Results expressed are triplicate mean ± SD with different superscript letters on each column signifying significant differences (*p* < 0.05). TFC, total flavonoid content; TPC, total phenolic content; ABTS, 2,2-azinobis (3-ethyl-benzothiazoline-6-sulfonic acid) antioxidant capacity; RMP, raw mopane worm flour; UMP, ultrasonicated mopane worm flour; FMP, fermented mopane worm flour; ROFSP, raw orange-fleshed sweet potato flour; UOFSP, ultrasonicated orange-fleshed sweet potato flour; FOFSP, fermented orange-fleshed sweet potato flour.

### 3.4 Techno-functional properties of raw and processed flours

Techno-functional properties describe the behavior of ingredients during preparation and cooking (Awuchi et al., 2019). It also provides some insights about how the final product may taste, feel, and look. The techno-functional properties of raw and processed flours are presented in Table 4. The OAC, which is the sample’s ability to absorb or hold water, increased significantly (*p* < 0.05) in MP flour after fermentation and ultrasonication, and this is desirable in food applications because it contributes to improved mouthfeel and retains the flavor of food products (Awuchi et al., 2019). OAC is related to the ability of the sample’s protein to attract and bind fat (Kasaye et al., 2018), meaning high-protein-containing food materials are more likely to absorb more oil. As a result, FMP and FOFSP exhibited higher OAC, probably due to increased protein content observed in Table 2. Fermentation also resulted in significant (*p* < 0.05) reduction in the WAC of both MP and OFSP flour. On the contrary, ultrasonic treatment significantly (*p* < 0.05) reduced the WAC of OFSP and increased that of MP flour. Generally, WAC relates to the hydrophilic groups of the sample’s carbohydrate and protein, which affect its water retention capacity (Farrokhi et al., 2025). The increase in WAC ultrasonicated flour is often associated with the generation of holes and microscopic channels, which expose hydrophilic binding sites, leading to the accumulation of more water molecules (Zheng et al., 2024). The ability of starch to absorb water and swell under a specific hydrothermal condition is known as swelling power (SP) (Jia et al., 2023). Fermentation and ultrasonication were observed to significantly (*p* < 0.05) reduce the SP of MP flour, while increments were only observed in FOFSP flour. The increase in SP of fermented OFSP was previously reported (Kewuyemi et al., 2025) and ascribed to the breakdown of proteins surrounding starch granules during fermentation, which restricts the swelling of starch by forming a barrier for water to penetrate it (Park et al., 2025). The reduction in SP of MP flour led to an increase in solubility. The low SP accompanied by high solubility is an indication of the weak forces of association between the starch granules and other constituents (Odey and Lee, 2020), attributed to lower or no starch content in MP. This is advantageous in the production of bakery and confectionery products (Dereje et al., 2020). The solubility of OFSP flour was significantly (*p* < 0.05) reduced after fermentation and increased after ultrasonication. It has been reported that ultrasonic treatment improves the solubility of food samples by facilitating the release of amylose chains, which are more susceptible to disruption due to lower structural integrity compared to amylopectin. Moreover, high solubility is crucial for the development of gluten-free bread, which helps improve dough texture and cohesion (Farrokhi et al., 2025).

**Table 4:** Techno-functional properties of raw and processed MP and OFSP flours.

Flours	OAC (g/g)	WAC (g/g)	SP (85 °C, g/mL)	Solubility (%)
Mopane worm				
RMP	0.08^a^ ± 0.00	0.16^bc^ ± 0.01	2.85^b^ ± 0.07	19.00^a^ ± 0.00
UMP	0.10^b^ ± 0.01	0.17^c^ ± 0.01	2.64^a^ ± 0.06	23.50^b^ ± 0.71
FMP	0.11^c^ ± 0.00	0.15^ab^ ± 0.00	2.58^a^ ± 0.06	26.50^c^ ± 0.71
Orange-fleshed sweet potato				
ROFSP	0.10^a^ ± 0.00	0.17^c^ ± 0.01	3.31^b^ ± 0.08	52.50^b^ ± 0.71
UOFSP	0.10^a^ ± 0.00	0.14^a^ ± 0.00	2.42^a^ ± 0.01	60.50^c^ ± 0.71
FOFSP	0.11^b^ ± 0.00	0.15^ab^ ± 0.01	4.19^c^ ± 0.01	32.00^a^ ± 1.41

Results expressed are triplicate mean ± SD with different superscript letters on each column signifying significant differences (*p* < 0.05). OAC, oil absorption capacity; WAC, water absorption capacity; SP, swelling power; RMP, raw mopane worm flour; UMP, ultrasonicated mopane worm flour; FMP, fermented mopane worm flour; ROFSP, raw orange-fleshed sweet potato flour; UOFSP, ultrasonicated orange-fleshed sweet potato flour; FOFSP, fermented orange-fleshed sweet potato flour.

### 3.5 Selected nutritional and health-promoting properties of processed blends

The nutritional composition of MP and OFSP blends is presented in Table 5. The moisture content of all the blends ranged from 8.14 to 9.84%. The addition of 55% MP flour in both processed blends increased their ash content, which could be attributed to the higher ash content of the mopane worm (Table 2). The increase in OFSP flour resulted in different observations between fermented and ultrasonicated blends. In ultrasonicated blends, there was a significant (*p* < 0.05) reduction in the ash content correlated with the increase in OFSP flour, while opposite observations were noted in fermented blends. The addition of more OFSP flour in ultrasonicated blends tended to reduce their fiber content. The increase in the fiber content of fermented blends could be due to the high resistant starch found in fermented OFSP (Kewuyemi and Adebo, 2024). According to the Food Nutrition Board of the Institute of Medicine of the National Academies and the American Association of Cereal Chemists, RS is defined as a form of dietary fiber that is not easily digested compared to typical starch, with similar properties as dietary fiber (Raigond et al., 2015). As anticipated, the increase in MP flour positively correlated with the protein content of the blends due to the higher protein content embedded in MP than OFSP flour (Table 2). These findings are in agreement with the previously reported study (Vanqa, 2022), wherein the addition of MP in wheat flour increased the protein content of the blends. Moreover, the highest protein was observed in fermented blends compared to their ultrasonicated counterparts, directly reflecting Table 2 observations, a decrease and an increase in protein content of ultrasonicated and fermented flours, respectively.

**Table 5:** Selected nutritional composition of fermented and ultrasonicated MP and OFSP flour blends.

Sample	Moisture (%)	Ash (%)	Fiber (%)	Protein (%)
UMP – 60	9.29^a^ ± 0.13	6.75^ab^ ± 0.04	4.42^ab^ ± 0.08	32.06^b^ ± 0.58
UMP – 55	8.98^a^ ± 1.24	6.21^a^ ± 0.02	4.11^ab^ ± 0.91	32.13^b^ ± 0.66
UMP – 45	9.84^a^ ± 0.01	6.83^ab^ ± 0.12	3.22^a^ ± 0.16	27.80^a^ ± 0.59
FMP – 60	8.14^a^ ± 0.30	7.29^b^ ± 0.47	3.67^ab^ ± 0.37	36.84^c^ ± 0.04
FMP – 55	8.24^a^ ± 0.22	6.83^ab^ ± 0.28	4.62^c^ ± 0.55	34.56^b^ ± 0.07
FMP – 45	8.72^a^ ± 0.12	8.35^c^ ± 0.47	4.90^b^ ± 0.33	29.97^a^ ± 0.39

Results expressed are triplicate mean ± SD with different superscript letters on each column signifying significant differences (*p* < 0.05). UMP – 60, ultrasonicated 60% MP and 40% OFSP blend; UMP – 55, ultrasonicated 55% MP and 45% OFSP blend; UMP – 45, ultrasonicated 45% MP and 55% OFSP blend; FMP – 60, fermented 60% MP and 40% OFSP blend; FMP – 55, fermented 55% MP and 45% OFSP blend; FMP – 45, fermented 45% MP and 55% OFSP blend.

The health-promoting properties of the investigated blends are shown in Table 6. As observed, the highest TFC (5.16 mgQE/g) and TPC (2.76 mgGAE/g) were found in FMP – 60 (fermented 60% MP and 40% OFSP blend). The increased TFC and TPC amounts after fermenting MP and OFSP as demonstrated in Table 3, present a good explanation for these findings. The increase in health-promoting properties such as TFC and TPC in fermented products is due to the release of bound phenolics prompted by enzymatic microbial activities during fermentation (Mudau and Adebo, 2025). The reduction in MP flour was observed to significantly (*p* < 0.05) reduce the TFC in fermented blends, and increase the TPC of ultrasonicated blends, showcasing that each processing technique offers unique modifications in the substrate’s phytochemical profile. Despite non-significant observations (*p* > 0.05) in the ABTS of investigated blends, the highest ABTS was observed in sample UMP – 60 (61.03%), followed by sample FMP – 45 (60.86%). A similar trend where the sample’s ABTS increases after fermentation and ultrasonication has been previously reported (Maleke et al., 2024; Mudau and Adebo, 2025), demonstrating that microbial activity and ultrasonic cavitation can catalyze the release or generation of soluble bioactive components with free radical scavenging potential. It is evident that food ingredients with high levels of health-promoting properties possess significant potential in managing and preventing most cardiovascular diseases (Saeed et al., 2025), which makes fermented and ultrasonicated MP and OFSP flour blends relevant for functional food development.

**Table 6:** Health-promoting properties of fermented and ultrasonicated MP and OFSP flour blends.

Flours	TFC (mgQE/g)	TPC (mgGAE/g)	ABTS (% inhibition)
UMP – 60	3.83^a^ ± 0.25	2.66^b^ ± 0.02	61.03^a^ ± 10.69
UMP – 55	4.48^bc^ ± 0.23	2.05^a^ ± 0.24	52.93^a^ ± 4.25
UMP – 45	4.21^ab^ ± 0.28	1.90^a^ ± 0.11	59.78^a^ ± 7.97
FMP – 60	5.16^c^ ± 0.21	2.76^a^ ± 0.12	59.20^a^ ± 6.00
FMP – 55	4.99^ab^ ± 0.27	2.48^a^ ± 0.30	55.85^a^ ± 1.82
FMP – 45	5.06^ab^ ± 0.51	2.45^a^ ± 0.21	60.86^a^ ± 6.26

Results expressed are triplicate mean ± SD with different superscript letters on each column signifying significant differences (*p* < 0.05). TFC, total flavonoid content; TPC, total phenolic content; ABTS, 2,2-azinobis (3-ethyl-benzothiazoline-6-sulfonic acid) antioxidant capacity; UMP – 60, ultrasonicated 60% MP and 40% OFSP blend; UMP – 55, ultrasonicated 55% MP and 45% OFSP blend; UMP – 45, ultrasonicated 45% MP and 55% OFSP blend; FMP – 60, fermented 60% MP and 40% OFSP blend; FMP – 55, fermented 55% MP and 45% OFSP blend; FMP – 45, fermented 45% MP and 55% OFSP blend.

### 3.6 *In vitro* starch digestibility of processed blends

The RDS (digested within 20 min), SDS (digested between 20 and 120 min), RS (undigested after 120 min), and TDS of all investigated blends are shown in Table 7. The RDS of ultrasonicated blends decreased as the proportion of OFSP flour increased, whereas fermented blends exhibited the opposite trend. This reduction in RDS of ultrasonicated blends is related to reduced risks of high blood glucose levels, vital for regulating type 2 diabetes (Lehmann and Robin, 2007). The increase in RDS in fermented blends may be ascribed to the activation of digestive enzymes during fermentation (Kewuyemi and Adebo, 2024), which can enhance starch hydrolysis. Ultrasonicated blends exhibited lower SDS compared to fermented blends. The SDS of UMP – 45 and FMP – 45 were 1.93 g/100 g and 3.12 g/100 g, respectively. A significant (*p* < 0.05) reduction in SDS of ultrasonicated blends as the proportion of OFSP increases. This is desirable, suggesting that ultrasonic treatment encourages resistance to starch hydrolysis, prolonging the digestion period (Wang et al., 2022). Moreover, it is well known that ultrasonic treatment induces disruptions in starch granules, rendering them more resistant to enzymatic degradation (Zhu, 2015; Yang et al., 2025). The addition of more OFSP flour tended to increase the SDS of processed blends, owing to its naturally larger starch granules, which often require more time for them to be completely hydrolyzed (Ketnawa et al., 2019). Statistically, there were no significant differences (*p* > 0.05) in the RS of all processed blends, but interesting findings were observed in fermented blends due to a slight increase in RS, positively correlating with the addition of more OFSP flour, known for comprising high RS content (Kewuyemi and Adebo, 2024). RS is regarded as a prebiotic component because of its ability to enhance bowel functioning and provide good stool output (Shen et al., 2017; Lončarević et al., 2021). The TDS of ultrasonicated blends ranged from 37.15 to 41.45 g/100 g compared to 37.52–47.76 g/100 g of fermented blends. In fact, there was an increase in TDS of processed blends prompted by a reduced proportion of MP flour, but ultrasonicated blends exhibited higher values compared to their fermented counterparts. Lower TDS in ultrasonicated blends shows that this processing technique can lower the digestion and absorption of starch, leading to various health benefits such as regulating glucose metabolism and glycemic index (Singh et al., 2010; Giuberti and Gallo, 2018; Bello-Perez et al., 2020). It is also worth stating that the addition of OFSP flour contributed to increased TDS among processed blends due to a higher amount of easily digestible sugars embedded in OFSP (da Silva et al., 2017) than MP worm flour.

**Table 7:** *In vitro* starch digestibility of fermented and ultrasonicated MP and OFSP flour blends.

Flours	RDS (g/100 g)	SDS (g/100 g)	RS (g/100 g)	TDS (g/100 g)
UMP – 60	36.34^b^ ± 0.55	1.01^a^ ± 0.98	39.94^a^ ± 0.55	37.15^a^ ± 0.33
UMP – 55	35.96^ab^ ± 0.15	1.22^a^ ± 0.15	38.76^a^ ± 1.06	37.53^a^ ± 0.53
UMP – 45	35.79^ab^ ± 0.84	1.93^ab^ ± 1.33	38.79^a^ ± 1.29	41.43^b^ ± 1.77
FMP – 60	34.57^a^ ± 1.02	1.98^ab^ ± 1.10	39.82^a^ ± 1.28	37.52^a^ ± 0.91
FMP – 55	35.65^ab^ ± 1.14	1.53^ab^ ± 1.07	40.03^a^ ± 0.27	40.36^b^ ± 0.35
FMP – 45	38.57^c^ ± 1.02	3.12^b^ ± 0.72	40.14^a^ ± 0.92	47.76^c^ ± 0.54

Results expressed are triplicate mean ± SD with different superscript letters on each column signifying significant differences (*p* < 0.05). RDS, rapidly digestible starch; SDS, slowly digestible starch; RS, resistant starch; TDS, total digestible starch; UMP – 60, ultrasonicated 60% MP and 40% OFSP blend; UMP – 55, ultrasonicated 55% MP and 45% OFSP blend; UMP – 45, ultrasonicated 45% MP and 55% OFSP blend; FMP – 60, fermented 60% MP and 40% OFSP blend; FMP – 55, fermented 55% MP and 45% OFSP blend; FMP – 45, fermented 45% MP and 55% OFSP blend.

### 3.7 Pasting properties of processed blends

The pasting properties describe changes that occur in food when heat is applied in the presence of water, and these changes affect the texture, digestibility, and end-use of food products (Ocheme et al., 2018). Table 8 shows the pasting properties of processed MP and OFSP flour blends. There was an increase in the peak viscosity of both fermented and ultrasonicated blends as the amount of OFSP increases, but significant (*p* < 0.05) observations were found only in fermented blends. Moreover, the peak viscosity of ultrasonicated blends was lower compared to that of fermented blends. For instance, UMP – 60 had a peak viscosity of 43.18 cP, compared to 92.70 cP of FMP – 60. According to Vela et al. (2024), high-frequency sound waves produced during ultrasonication can break long macromolecular chains into shorter chains, leading to low viscosity profiles. High peak viscosities in fermented samples may be attributed to the breaking down of starch granules, leading to poor structural rigidity and increased swelling (Yuliana et al., 2023), and it may also be related to high WAC and SP observed in FOFSP flour (Table 4). No significant observations (*p* > 0.05) were noted in the peak time amongst processed blends, but the lowest was found in UMP – 45 (28.80 min). Lower peak time is often associated with energy efficiency upon processing, which suggests that less energy during cooking may be required. The SVP and SVT followed a similar trend, and the lowest viscosities were observed in ultrasonicated blends. Higher setback viscosities in fermented blends suggest a higher probability of retrogradation due to the formation of a gel structure capable of reorienting itself (Mudau and Adebo, 2025). The breakdown viscosity reflects how stable the peak viscosity would be during processing (Awolu, 2017). Low breakdown viscosity among fermented blends is an indication of an increased blend’s ability to withstand heating and shear stress upon cooking (Ocheme et al., 2018), probably attributed to higher protein and fiber contents, which are capable of restricting the swelling of starch granules, by competing with the starch granules for available water, leading to decreased viscosities (Chinma et al., 2023). No significant differences (*p* > 0.05) were observed between the FV and the PAT of all investigated blends, but ultrasonicated blends exhibited relatively higher FV, which is a desirable characteristic, especially for food products where thickness and stability are key requirements (Adeyanju et al., 2025). Fermentation and ultrasonication offered varying modifications in the pasting properties of MP and OFSP blends, and these findings provide valuable insights about possible applications of each processing technique for product development using MP and OFSP blends.

**Table 8:** Pasting properties of fermented and ultrasonicated MP and OFSP flour blends.

Sample	PV (cP)	PT (min)	BV (cP)	SVP (cP)	SVT (cP)	FV (cP)	PAT (°C)
UMP – 60	43.18^a^ ± 1.53	31.95^a^ ± 1.20	3.07^a^ ± 1.63	5.97^ab^ ± 2.56	46.08^a^ ± 2.65	45.87^a^ ± 3.63	95.10^a^ ± 0.00
UMP – 55	48.23^a^ ± 1.84	33.15^a^ ± 0.07	1.01^a^ ± 0.35	8.73^ab^ ± 0.58	55.96^a^ ± 2.78	49.28^a^ ± 8.45	95.10^a^ ± 0.00
UMP – 45	60.47^a^ ± 3.79	26.80^a^ ± 8.49	15.55^b^ ± 3.73	2.62^a^ ± 2.53	47.54^a^ ± 4.99	43.30^a^ ± 0.00	94.15^a^ ± 1.34
FMP – 60	92.70^a^ ± 10.38	33.15^a^ ± 0.07	0.88^a^ ± 0.29	9.38^ab^ ± 3.60	101.36^a^ ± 14.48	43.30^a^ ± 0.00	95.10^a^ ± 0.00
FMP – 55	137.55^b^ ± 12.37	33.10^a^ ± 0.00	1.19^a^ ± 0.47	16.30^bc^ ± 4.92	152.65^b^ ± 7.00	43.30^a^ ± 0.00	95.10^a^ ± 0.00
FMP – 45	325.00^c^ ± 24.89	33.15^a^ ± 0.00	2.04^a^ ± 2.88	25.67^c^ ± 8.72	297.30^c^ ± 13.29	43.30^a^ ± 0.00	95.10^a^ ± 0.00

Results expressed are triplicate mean ± SD with different superscript letters on each column signifying significant differences (*p* < 0.05). PV, peak viscosity; PT, peak time; BV, breakdown viscosity; SVP, setback viscosity from peak; SVT, setback viscosity from trough; FV, final viscosity; PAT, pasting temperature; UMP – 60, ultrasonicated 60% MP and 40% OFSP blend; UMP – 55, ultrasonicated 55% MP and 45% OFSP blend; UMP – 45, ultrasonicated 45% MP and 55% OFSP blend; FMP – 60, fermented 60% MP and 40% OFSP blend; FMP – 55, fermented 55% MP and 45% OFSP blend; FMP – 45, fermented 45% MP and 55% OFSP blend.

### 3.8 Thermal properties of processed blends

Thermal properties determined by DSC are critical for understanding the sample’s thermal behavior and heat transfer responses (Falade and Christopher, 2015; Raigar and Mishra, 2015). Particularly in food, these properties relate to the characteristics of starch, such as granule size and degree of crystallinity, which influence its gelatinization behavior (Chinma et al., 2022b). The thermal properties of processed blends based on onset temperature (T_o_), peak temperature (T_p_), conclusion temperature (T_c_), and enthalpy change (∆H) are presented in Table 9. The T_o_ of samples ranged from 86.77 to 112.50 °C in fermented blends, and 97.37–107.99 °C in ultrasonicated blends, and this temperature reflects the preliminary phase of temperature transition, such as melting (Adeyanju et al., 2025). The replacement of 60% MP flour in all processed blends with 50 and 45% resulted in a significant (*p* < 0.05) increase in the T_o_, implying that blends with 60% MP flour required a lower temperature to start gelatinization. No significant (*p* > 0.05) differences were observed in T_p_, and T_c_ of blends, but ultrasonicated blends exhibited higher T_p_. Higher thermal properties, including T_p_ were previously reported (Yang et al., 2019) in ultrasonicated starch-containing food matrix, due to the termination of weak crystalline structures in starch granules caused by ultrasonic cavitation. A slight reduction in T_c_ of fermented blends may be attributed to damage caused by the actions of mesophilic lactic acid bacteria on the crystal structure (resulting in a less thermally stable and ordered crystalline structure) (Adeyanju et al., 2025) of OFSP starch within the blends. Mudau and Adebo (2025) asserted that the nature of protein-starch structure and their interactions within the food matrix can significantly affect the thermal transitions of samples, especially when protein-rich food material is involved. Despite non-significant observations (*p* > 0.05), the ∆H change of ultrasonicated blends was lower, ranging from 3,743.43 to 3,899.39 Jg^−1^, compared to 4,222.58–4,309.48 Jg^−1^ of their fermented blends. Enthalpy is critical for controlling processing and storage conditions in the food production chain, and it indicates the amount of energy required to cause thermal transitions (Adeyanju et al., 2025; Zhou, 2025). Low ∆H change in fermented blends could be due to disruption of the starch crystalline structure in OFSP flour, which often requires less energy to induce thermal transitions.

**Table 9:** Thermal properties of fermented and ultrasonicated MP and OFSP flour blends.

Sample	T_O_ (°C)	T_P_ (°C)	T_C_ (°C)	∆H (Jg^−1^)
UMP – 60	97.37^ab^ ± 0.72	115.60^a^ ± 1.19	139.69^a^ ± 2.12	3,842.67^a^ ± 324.63
UMP – 55	103.34^b^ ± 2.11	115.98^a^ ± 2.78	141.33^a^ ± 25.08	3,899.39^a^ ± 262.77
UMP – 45	107.99^b^ ± 4.94	115.92^a^ ± 0.56	136.55^a^ ± 2.36	3,743.43^a^ ± 108.30
FMP – 60	86.77^a^ ± 13.19	113.82^a^ ± 0.94	145.38^a^ ± 18.54	4,222.58^a^ ± 34.18
FMP – 55	112.50^b^ ± 1.81	112.72^a^ ± 1.87	136.89^a^ ± 2.22	4,309.48^a^ ± 83.01
FMP – 45	102.55^b^ ± 6.77	113.55^a^ ± 1.83	135.05^a^ ± 4.99	4,308.42^a^ ± 416.29

Results expressed are triplicate mean ± SD with different superscript letters on each column signifying significant differences (*p* < 0.05). T_o_, onset temperature; T_p_, peak temperature; T_c_, conclusion temperature; ∆H, enthalpy change; UMP – 60, ultrasonicated 60% MP and 40% OFSP blend; UMP – 55, ultrasonicated 55% MP and 45% OFSP blend; UMP – 45, ultrasonicated 45% MP and 55% OFSP blend; FMP – 60, fermented 60% MP and 40% OFSP blend; FMP – 55, fermented 55% MP and 45% OFSP blend; FMP – 45, fermented 45% MP and 55% OFSP blend.

## 4 Conclusion

This study shows that fermentation and ultrasonication can modify the nutritional composition, health-promoting properties, functionality, and *in vitro* starch digestibility of MP and OFSP flour blends in different ways. Fermenting blends using mesophilic lactic acid bacteria showed improved ash, protein, TFC, and TPC, while ultrasonicated blends exhibited better starch digestibility profiles. Thermal properties were in favor of fermented blends as demonstrated by lower temperature transitions. Interestingly, both fermentation and ultrasonication induced beneficial modifications in the pasting characteristics of blends, with optimal findings recorded in ultrasonicated blends. Higher peak viscosity and lower breakdown viscosity found in fermented blends are ideal for the development of thickened dysphagia diets, while lower peak time in ultrasonicated blends may signify energy efficiency during processing. Therefore, both processing techniques could offer distinct improvements and beneficiation possibilities in the properties of MP and OFSP blends, which could necessitate the development of novel and special foods (e.g., baked products, snacks, etc.) with functional ingredients. Other analyses such as shelf life, other antioxidant activity tests, chromatographic profiling of the health benefits and compositional analysis of subsequent products could be explored in future studies.

## Author’s note

A Dutch provisional patent application has been submitted with application number 2040981.

## Data Availability

The raw data supporting the conclusions of this article will be made available by the authors, without undue reservation.
